# A general way for quantitative magnetic measurement by transmitted electrons

**DOI:** 10.1038/srep18489

**Published:** 2016-01-04

**Authors:** Dongsheng Song, Gen Li, Jianwang Cai, Jing Zhu

**Affiliations:** 1 National Center for Electron Microscopy in Beijing, Key Laboratory of Advanced Materials (MOE) and The State Key Laboratory of New Ceramics and Fine Processing, School of Materials Science and Engineering, Tsinghua University, Beijing 100084, China; 2 Beijing National Laboratory for Condensed Matter Physics, Institute of Physics, Chinese Academy of Sciences, Beijing 100190, China

## Abstract

EMCD (electron magnetic circular dichroism) technique opens a new door to explore magnetic properties by transmitted electrons. The recently developed site-specific EMCD technique makes it possible to obtain rich magnetic information from the Fe atoms sited at nonequivalent crystallographic planes in NiFe_2_O_4_, however it is based on a critical demand for the crystallographic structure of the testing sample. Here, we have further improved and tested the method for quantitative site-specific magnetic measurement applicable for more complex crystallographic structure by using the effective dynamical diffraction effects (general routine for selecting proper diffraction conditions, making use of the asymmetry of dynamical diffraction for design of experimental geometry and quantitative measurement, etc), and taken yttrium iron garnet (Y_3_Fe_5_O_12_, YIG) with more complex crystallographic structure as an example to demonstrate its applicability. As a result, the intrinsic magnetic circular dichroism signals, spin and orbital magnetic moment of iron with site-specific are quantitatively determined. The method will further promote the development of quantitative magnetic measurement with high spatial resolution by transmitted electrons.

The electron energy-loss magnetic chiral dichroism signal of magnetic materials was first detected in the transmission electron microscope by Schattschneider P. *et al.* in 2006^1^. In the last few years, rapid development of the EMCD technique has been made both in theory^2^,^3^,^4^,^5^,^6^ and experiment^7^,^8^,^9^,^10^,^11^. Combined with advanced characterization methods of transmission electron microscope (TEM), the EMCD technique shows a promising prospect in the fields of nanomagnetism^12^, multiferroics^13^ and spintronics^14^. By applying the sum rules to EMCD signals, the quantitative magnetic parameters are obtained^5^,^15^. However, the electron-based EMCD technique rather than X-ray, accompanied with the remarkable electron dynamical diffraction effects in periodic crystal structure^2^,^13^,^16^,^17^, leads to the additional dynamical coefficients that depend on the dynamical diffraction conditions in the EMCD sum rules^5^,^15^, making the extraction of magnetic parameters more complicated than XMCD technique.

The experimentally proven technique of site-specific EMCD was first used for Ni_2_MnSn^18^ in 2012 and further developed to obtain rich quantitative magnetic information from the same kind of atoms sited at nonequivalent crystallographic planes in NiFe_2_O_4_^13^ ferrite with inverse spinel structure in 2013, which cannot be achieved by XMCD technique^13^. However, site-specific EMCD is still a complex technique, and include the following aspects: (1) all the attention along with EMCD technique should be carefully paid, such as the experiment setup of diffraction geometry^8^,^9^,^10^,^19^,^20^,^21^, the effect of asymmetry of dynamical diffraction effects^10^,^22^,^23^,^24^,^25^,^26^,^27^, low signal-noise ratio (SNR); (2) extra critical requirements from site-specific EMCD itself have to be met, such as the proper dynamical diffraction conditions, in the face of the restrictions on crystallographic structure^13^; (3) until now, the site-specific EMCD technique have only been successfully applied to Ni_2_MnSn^18^ and NiFe_2_O_4_^13^ as reported. For other more complex structure of magnetic oxides, such as YIG, it may fail to achieve the magnetic measurement. Therefore, successfully applying the EMCD technique for each particular magnetic material and quantitatively achieving a reliable and accurate measurement of site-specific magnetic information require a deep and comprehensive understanding of the EMCD technique and dynamical diffraction effects in terms of both theory and experiment.

In this work, we aim to establish an improved method of quantitative site-specific magnetic measurement of magnetic materials applicable for more complex crystallographic structure by making full use of the effective dynamical diffraction effects, which include the analysis of crystallographic structure for proper dynamical diffraction conditions, calculations of dynamical diffraction effects, and especially the construction and optimization of diffraction geometry with the consideration of asymmetry. Yttrium iron garnet (YIG), a typical magnetic insulator oxide with complex crystallographic and magnetic structure, is used to show the good applicability of our method in the experiments. The intrinsic magnetic circular dichroism (MCD) signals and rich magnetic parameters for Fe ions of different sites are quantitatively determined at last.

## Results

### Crystallographic structure

YIG (Y_3_Fe_5_O_12_) has a ferrimagneitc and garnet structure with a lattice parameter of a = 12.376 Å. Yttrium ions are located at the dodecahedral sites, and iron ions are located at octahedral (oct) and tetrahedral (tet) sites with oxidation states +3, respectively. The number ratio of octahedral Fe and tetrahedral Fe is 2:3. The ferrimagnetic order occurs below the Curie temperature, *T*_*c*_ = 539 *K*, and consists of the ferromagnetic alignment of the magnetic moments of octahedral and tetrahedral Fe ions in alternate {111} planes being antiparallel. The magnetic structure can be written as Y_3_ [Fe^3+^_2_, ↓]_oct_ [Fe^3+^_3_, ↑ ]_tet_ O_12_.

**Figure 1:**
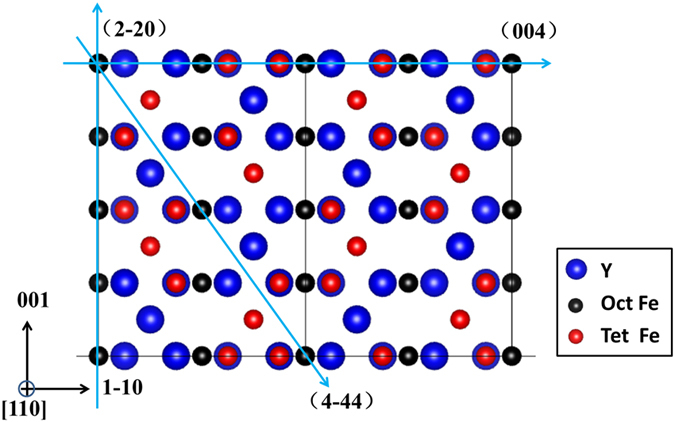
Crystallographic structure of YIG . Views of the unit cell along the [110] crystallographic direction. The (004), (2-20), (4-44) planes are highlighted by the light blue lines.

The projection of the YIG unit cell along the [110] direction is shown in Fig. 1 (not including oxygen atoms). The most basic requirement for resolving magnetic structure with different crystallographic occupied sites (such as YIG) is to get two kinds of diffraction conditions to enhance the EMCD signals of the same magnetic element at different sites respectively^13^. Obviously, the octahedral Fe and tetrahedral Fe are easily separated in several planes, but the yttrium and tetrahedral Fe always overlap together. This will increase the crystal potential of planes with tetrahedral Fe, making the EMCD signals of octahedral Fe difficult to be enhanced. The (004), (2-20) and (4-44) planes indicated in Fig. 1 in the manuscript are taken as the example to analyze the enhancement of dynamical diffraction effects under the planar channeling conditions. The (004) planes are with (4Y + 4tetFe + 4octFe)/(2Y + 2tetFe) alternately arranged. The heavy atomic planes contain both tetrahedral and octahedral Fe, so the separation of different crystallographic sites is not good and the enhancement of tetrahedral Fe is very weak. The (2-20) planes are with (Y + tetFe)/(Y + tetFe)/(Y + tetFe)/(2octFe) alternately arranged. Although it gives a good separation, the four planes have similar crystal potential; hence the enhancement is also weak. It turns out that only the (4-44) planes with (3Y + 3tetFe)/(2octFe) alternately arranged totally separate the tetrahedral and octahedral Fe and have a reasonable difference in crystal potential, leading to the significant enhancement of tetrahedral Fe. However, we don’t find another planar channeling condition for octahedral sites enhanced like NiFe_2_O_4_, for which the octahedral and tetrahedral Fe are enhanced under the (004) and (2-20) planar channeling conditions, respectively^13^. The different structure of garnet and spinel results in this difference. Meanwhile, it also shows the strong dependence and restrictions of the site-specific EMCD technique on the crystallographic structure.

### Simulation of EMCD signals

**Figure 2:**
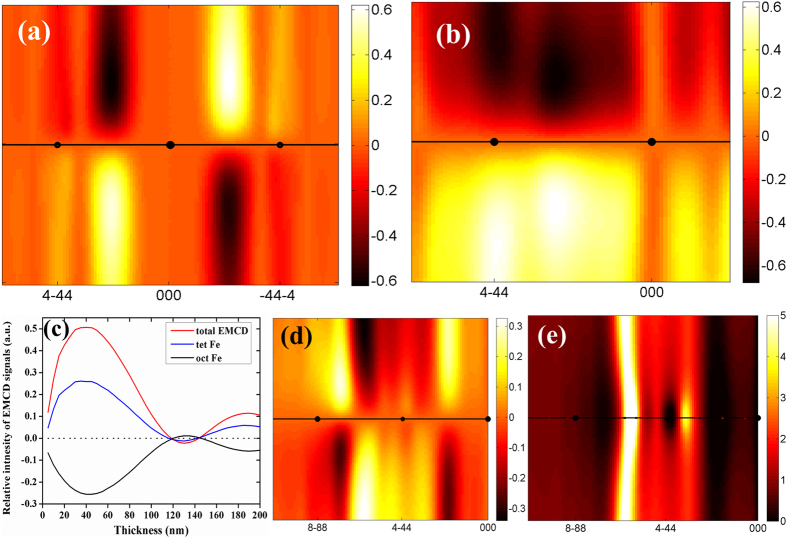
Results of relative intensity of simulated EMCD signals by dynamical diffraction calculations at 300 kV with the thickness of 45 nm. (**a**,**b**) distribution of EMCD signals in the diffraction plane with (4-44) planes strongly excited under the three-beam and two-beam case, respectively; (**c**) EMCD signals as a function of thickness (taken from Thales circle with a diameter of 0.25**g**_4-44_ in the diffraction plane under two-beam case). Blue, red and black lines are corresponding to total, tetrahedral and octahedral EMCD signals, respectively; (**d**) distribution of EMCD signals with (8-88) planes strongly excited; (**e**) distribution of the ratio of dynamical coefficients from octahedral and tetrahedral sites with (8-88) planes strongly excited.

The simulation of EMCD signals through quantitative calculations of dynamical diffraction effects is conducted^28^ (see supplementary information for details of calculations). Only the excitation of (4-44) planes leads to a strong intensity of EMCD signals, which is almost five to ten times stronger than those of others (see supplementary information), consistent with the qualitative analysis. The distribution of EMCD signals in the reciprocal space under the most commonly used two-beam and three-beam diffraction geometry with (4-44) planes excited for YIG are displayed in Figs 2(a) and 2(b) (we use the dynamical coefficients to represent the distribution of relative intensity of measured EMCD signals, see eq. (9) in Methods). The strong overall EMCD signals in the diffraction plane can provide a large reasonable collecting area and high SNR with finite collection angles, which is favorable for quantitative measurements.

The quantitative description of EMCD signals by dynamical diffraction effects is necessary for the optimization of diffraction geometry and interpretation of experimental results. Moreover, it will also help us find the diffraction conditions for the enhancement of octahedral Fe of YIG. The interaction between fast electrons and magnetic materials includes elastic and inelastic scattering, and the dynamical diffraction effects depend on the direction of incident and outgoing beam, and the sample thickness. Therefore, by tuning these factors we may achieve the enhancement of octahedral sites. The relative intensity of simulated EMCD signals for (4-44) planes excited is shown in Fig. 2(c) as the function of thickness. Except for sign inversion at a thickness of about 130 nm, all other thicknesses are corresponding to the enhancement of tetrahedral Fe. But the thick sample will lead to higher background and lower SNR, which will introduce large error in the quantitative measurements. In addition, the relative intensity of measured EMCD signals at 130 nm is very weak and cannot be detected in this experiment. Therefore, the method by changing thickness is not suitable for YIG.

For quantitative EMCD technique, the precise control of diffraction conditions is critical for reliable EMCD signals and quantitative magnetic measurements, which makes the two-beam and three-beam diffraction geometry popular^10^. The incident angle for two-beam and three-beam case is θ_B_ and 0 (θ_B_ is the Bragg angle for YIG (4-44) planes), respectively. Another diffraction condition easy to be controlled is with 2θ_B_ incident to make (8-88) planes strongly excited. Therefore, the outgoing angle ranges from −2θ_B_ to 2θ_B_ corresponding to positions of (8-88) and (000) diffraction spots respectively in the reciprocal plane, providing a large area for detector positions. The simulation of relative intensity of EMCD signals for YIG with the (8-88) planes strongly excited in the diffraction plane is shown in Fig. 2(d). The signals vary at different positions caused by the different outgoing conditions. Moreover, the sign of the signals is inversed and the octahedral Fe is enhanced around the white (black) areas in the upper (lower) half plane as shown in Fig. 2(d). It can be deduced from the Fig. 2(e) that the area where the dynamical coefficients of octahedral site is larger than that of tetrahedral sites (ratio < 1), is corresponding to the enhancement of octahedral site. (see supplementary information for the analysis of octahedral enhancement). The intensity of EMCD signals are strong enough to be detected for quantitative measurements. All in all, by tuning the factors related to dynamical diffraction effects, we are always able to find the proper diffraction conditions to enhance the elements at different crystallographic sites.

Based on the above simulation, the diffraction conditions for experiments are determined. To precisely control the diffraction geometry, we use the Kikuchi lines to tilt the orientation of the sample for the pre-established incident conditions. Slight adjustment of beam tilting is also used to overcome the mechanical error of the TEM double tilt holder. The thickness at the area for spectra acquisition is 46.7 ± 1.7 nm (see Methods) and is almost corresponding to strongest EMCD signals as shown in Fig. 2(c). The detector positions are determined by moving the diffraction pattern with respect to the fixed entrance aperture of EELS spectrometer. However, it has been pointed out that area for spectra acquisition in the diffraction plane should not only be with high intensity of EMCD signals but also free from asymmetry^23^,^27^. Therefore, the optimization of detector positions for asymmetry is conducted to guarantee a reliable and pure EMCD signal (see supplementary information).

## Experimental results

**Figure 3:**
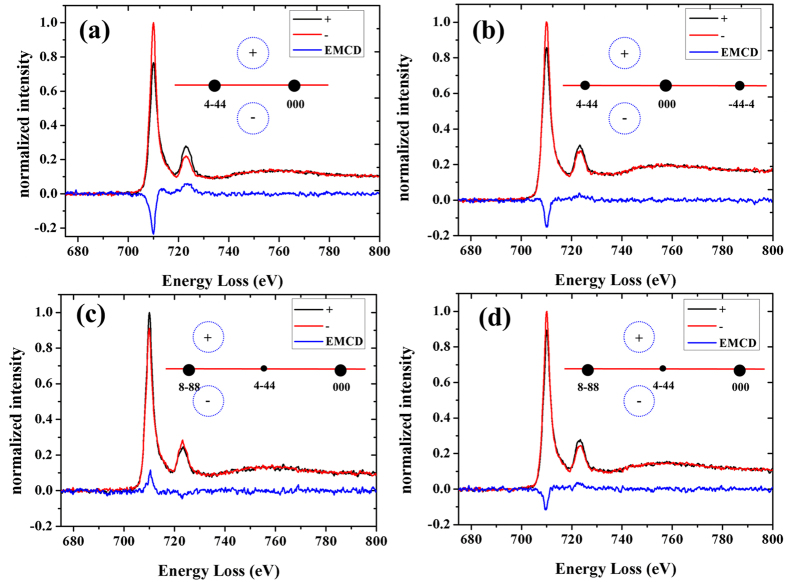
Experimental EMCD signals of Fe element in YIG under different diffraction conditions. (**a**,**b**) are the EMCD signals from two-beam and three-beam case, respectively; (**c**,**d**) are the EMCD signals with the octahedral and tetrahedral Fe enhanced under the incident angle of 2θ_B_, respectively; The black and red lines represent the EELS spectra from “+” and “−” positions, and the blue lines represents the EMCD signals. The schematic drawings in each figure briefly show the diffraction geometry and the blue circles represent the positions of entrance aperture.

The EMCD experiment consists of taking two EELS spectra at particular conjugate positions in the diffraction plane and the EMCD signal is the difference between them. Based on the established diffraction geometry and proper positions of collection aperture, the acquired EMCD signals of element Fe for YIG are shown in Fig. 3, and the corresponding diffraction geometry and detector positions are displayed in the illustrations. The experimental results are well coincided with the results of calculations and the intensity of EMCD signals under the two-beam case (Fig. 3(a)) is stronger than that under the three-beam case (Fig. 3(b)). When the incident angle is tilted to 2θ_B_, we acquire the EMCD signals at the positions with octahedral (Fig. 3(c)) and tetrahedral (Fig. 3(d)) Fe enhanced as predicted by the calculations of dynamical diffraction effects. As expected, the sign of signals is inversed in the experiments, showing the subtle control of EMCD signals by dynamical diffraction effects.

### Quantitative magnetic parameters

Although the EMCD signals of Fe element for YIG can be obtained under different diffraction conditions, the signals from different sites are still overlap together and cannot be separated directly. For the measured EMCD signals consist of magnetic contribution from each of the nonequivalent atomic site, each spectra for YIG at a certain dynamical diffraction condition can be generally expressed as the intrinsic MCD signals from octahedral Fe and tetrahedral Fe with different weighted coefficients as follows,


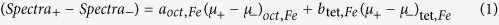


***Spectra***+ and ***Spectra***_***−***_ are EELS spectra from the conjugate positions; ***a*** and ***b*** are the weighted coefficients corresponding to the dynamical coefficients; (***μ***_+_***– μ***_***−***_) is the intrinsic MCD signal. To extract the MCD signals for octahedral and tetrahedral Fe of YIG, a series of experimental measured EMCD spectra are acquired under different dynamical diffraction conditions and the matrix form of equation (1) is as follows^29^,^30^,


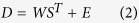


where 
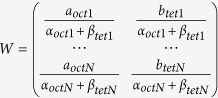
, N is the number of spectra, a, b, α, β are the dynamical coefficients as described in **Methods**.

**Figure 4:**
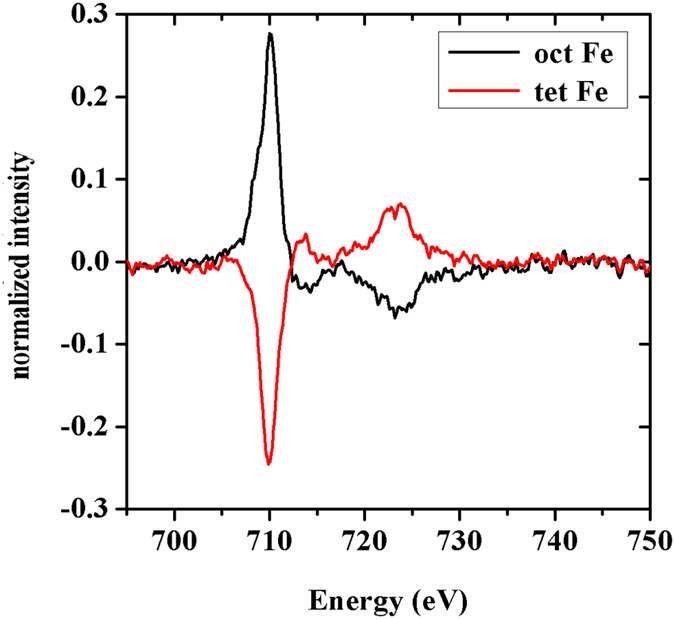
Intrinsic EMCD signals for octahedral (black) and tetrahedral (red) Fe of YIG.

The matrix ***D*** represents the experimental data with set of the mixed EMCD spectra. The matrix ***S*** represents the intrinsic MCD signal of octahedral and tetrahedral Fe. The matrix ***W*** is composed of dynamical coefficients from calculations, and ***E*** denotes the residual. Through least square fitting method, the optimal matrix ***S***^***T***^ including the pure MCD spectra for octahedral and tetrahedral sites is solved to fit the experimental data matrix ***D*** to the best level for which the sum of the square of the elements in matrix ***E*** is minimized. The optimal solution is ***S***^***T***^*** = (W***^***T***^***W)***^***−1***^***(W***^***T***^***D)***. The intrinsic MCD signals for octahedral and tetrahedral Fe of YIG is shown in Fig. 4. The different intensity of EMCD signals shows the different magnetic parameters for Fe in different crystal field. The opposite sign of EMCD signals for octahedral and tetrahedral Fe indicates the antiferromagnetic coupling between them. Please note that our method is also able to get the information of magnetic coupling between octahedral and tetrahedral Fe, which is not used throughout the entire process of signal extraction.

Applying the sum rules to the intrinsic MCD signals, the magnetic parameters of YIG are obtain as listed in Table 1. The errors for magnetic parameters are also estimated (see supplementary information). The sum rules used here is the formula of XMCD^31^,^32^ (eqs. (3) and (4)) rather than that of EMCD, which contains the dynamical coefficients^5^,^15^. This is because during signal extraction, the dynamical coefficients have already been considered and the intrinsic signals are free from the dynamical diffraction effects. We also compare the results with those from neutron diffraction and first principle calculation, though many of the parameters cannot be obtained by other magnetic characterization techniques. To further highlight our quantitative results, we use the macroscopic superconducting quantum interference device (SQUID) to measure hysteresis loop of the same YIG thin film that was used in TEM-EMCD measurement (see supplementary information). The macroscopic saturation magnetization is normalized and the total moment of the unit cell is 3.3 μ_B_, which is close to our EMCD results.


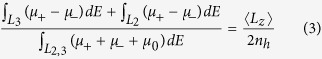



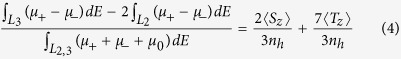


where <Sz>, <Lz>, <Tz> are respectively the ground-state expectation values of spin momentum, orbital momentum, and magnetic-dipole operators, while N_*h*_ is the number of *d* holes^31^,^32^.

## Discussion

The complicated dynamical diffraction effects have always been deemed to be a disadvantage of EMCD technique before. On the contrary, by using the effective dynamical diffraction effects, we realize the extraction of the intrinsic MCD signals free from the dynamical diffraction effects and rich magnetic parameters with site-specific for more complex crystallographic structure, which cannot be achieved by XMCD and other magnetic characterized technique. Besides, except for the feasibility for garnet with complex crystallographic and magnetic structure, the method is also possible for a wide range of ferromagnetic or ferromagnetic materials with simple or complicated crystallographic structure.

In summary, the improved method to achieve the quantitative site-specific magnetic measurement is well-established throughout the present paper with the example of YIG. A general routine to effectively tune the EMCD signals through dynamical diffraction effects is systematically claimed to make our method applicable to various magnetic materials with complex crystallographic structures. Consideration of asymmetry of dynamical diffraction effects is involved both in the optimization of experimental conditions and quantitative measurement. At last, the intrinsic MCD signal, spin and orbital magnetic moment of iron ions are finally quantitatively determined. Our method will further promote the development of quantitative magnetic measurement of the EMCD technique in the transmission electron microscope.

## Methods

### Sample information

The (111) YIG single crystalline thin film was epitaxially grown on (111) Gd_3_Ga_5_O_12_ (GGG) garnet substrates with small mismatch (0.05%) by liquid phase epitaxy method. The thickness of YIG is about 13.5 μm. The X-ray diffraction reflects the high crystalline quality of YIG and GGG. Magnetic hysteresis loop measurements performed on a Quantum Design superconducting quantum interference device magnetometer (SQUID) show that the YIG film is magnetically soft and almost isotropic in the film plane at room temperature with a saturated magnetization of 1.75 kG. These magnetic properties agree well with those of bulk YIG. The cross-sectional microscope sample was produced by conventional methods, which include mechanical thinning and low-angle Ar^+^ ion milling at different voltages to achieve electron transparency.

### Calculations of dynamical diffraction effects

The EELS spectra at conjugate positions of ‘+’ and ‘−’ in the diffraction plane could be written as follows^27^,


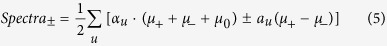



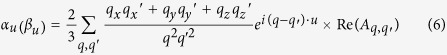



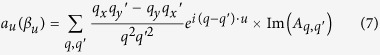


The terms related to dynamical diffraction effects is defined as *A*_*q,q*′_, including the thickness function and Bloch coefficients, and detailed definitions can be found in ref. 2. 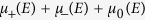 is the nonmagnetic signal and 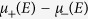 is the magnetic signal,***u*** represents the coordinates of different atoms at different positions in a unit cell, and it represents the octahedral site for a and α, tetrahedral sites for b and β. ***α***_***u***_ and ***a***_***u***_ are the dynamical coefficients for nonmagnetic and magnetic components, respectively.

For YIG, it can be expressed as,


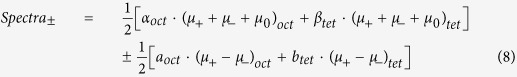


The calculations consist of two parts. First, the Bloch wave software^28^ was used to get the Bloch coefficients and momentum transfer based on the certain experimental diffraction conditions. Second, the dynamical coefficients in equation (6) and (7) are calculated with the code developed by ourselves. The relative intensity of measured EMCD signals for YIG is defined in equation (9) and is proportional to the ratio of dynamical coefficients, which is approximated that the intrinsic nonmagnetic signals 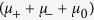 for octahedral and tetrahedral Fe are equal, and also the intrinsic magnetic signals 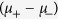.


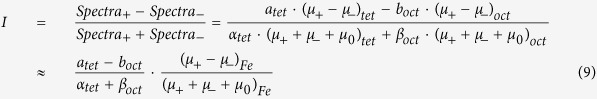


Thus, the relative intensity of EMCD signals is proportional to the dynamical coefficient 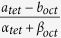, and we use it to represent the distribution of relative intensity of EMCD signals as shown in Fig. 2. As the intrinsic MCD signal of tetrahedral and octahedral sites are opposite, the sign of measured EMCD signals in the experiments is determined by the value of dynamical coefficients. The signal of Fe ions from crystallographic site that corresponds to larger coefficients is enhanced because it is corresponding to high excitation of signals at this site.

The calculations are conducted under the conditions of systematic reflection. To get the dynamical coefficients described in the manuscript, the (incident/outgoing) 5/5 beam case along the reflections axis were used, and experimental conditions including sample thickness, Laue circle center and detector position were input for the calculation procedure. The High-order Laue Zone (HOLZ) effects are not included in the calculations. The multi-beams dynamical eigenvalue equations are resolved by the *Blochwave* software provided by Dr. S. Löffler^28^. Then our homemade Matlab code is used to calculate the final dynamical coefficients. The calculation is based on the assumption of plane-wave illumination and point-like detection. The convergence of dynamical calculation^33^ is detailed discussed in the supplementary information.

### Thickness measurement

The thickness of the sample between 30 nm and 50 nm are corresponding to the strongest EMCD signals as shown in Fig. 2(c). The thickness at the probed area for spectra acquisition is 46.7 ± 1.7 nm, which is determined both by CBED (convergent beam electron diffraction) and low-loss EELS. Since the effective plasmon mean free path of inelastic scattering (λ) estimated through empirical formula has a large error of 10%, we first choose a thicker area (80 ~ 120 nm) for measurement by CBED (with an error of about 3%) to determine its actual thickness. Then, the low loss EELS is acquired on the microscope of FEI Titan 80-300 rather than JEOL 2010F at the same area where the absolute thickness has already been measured by CBED. At last, the mean free path is calibrated with the experimental conditions of FEI Titan 80-300. At last, the thickness of thin area for data acquisition, which is not easily determined by CBED, is calculated with low-loss EELS and calibrated λ. In the experiment, this thickness is neither too thin to bear the irradiation damage, nor too thick to result in the low SNR. More importantly, the Kikuchi lines are clear to be distinguished in the experiments for the adjustment of crystallographic orientation.

### Data acquisition and processing

The EELS and EMCD experiments were performed on the FEI Titan 80-300 operating at 300 kV, attached with a post-column Gatan Tridium system with the energy resolution of 0.7 eV. The microscope is extremely stable to ensure experimental conditions unchanged during the experiments. In the EMCD measurements, a nearly parallel beam (convergent angle <0.5 mrad) with probe size of around 50 nm in diameter is used to illuminate the sample. The tilt angle is about 9.4° to reach the 3-beam diffraction geometry from the [110] zone axis. By tilting the sample further in the perpendicular direction by a small angle of 0.3° according to the Kikuchi lines, the 2-beam diffraction geometry is obtained. The collection angle is about 3.4 mrad. The acquisition time of each spectrum is about several seconds for high SNR and irradiation damage can be negligible. The EELS data processing include pre-edge background subtraction and removal of plural scattering by Fourier-ratio deconvolution using a zero-loss modifier function in the Digital Micrograph software^34^. The ‘+’ and ‘−’ deconvoluted edges are normalized by the integration of the intensity in a post-edge window between 50 eV and 100 eV after the onset energy of the edge L3 to against the effects of asymmetry, based on an assumption that in the post-edge region the magnetic signal is negligible and only non-magnetic spectral components remain^26^. By subtracting the two spectra, the EMCD signals are obtained. The experiment of CBED was performed on the JEOL 2010F operating at 200 kV.

## Additional Information

**How to cite this article**: Song, D. *et al.* A general way for quantitative magnetic measurement by transmitted electrons. *Sci. Rep.* **6**, 18489; doi: 10.1038/srep18489 (2016).

## Supplementary Material

Supplementary Information

## Figures and Tables

**Table 1:** Magnetic parameters of YIG.

Magnetic parameters	EMCD (present work)	XMCD	Neutron diffraction	First principle calculation	Macro measurement (SQUID, present work)
m_L_/m_S_ (oct)	0.06 ± 0.02				
m_L_/m_S_ (tet)	0.08 ± 0.02				
m_L_ (oct)	0.28 ± 0.03				
m_L_ (tet)	0.31 ± 0.03				
m_S_ (oct)	4.5 ± 0.2				
m_S_ (tet)	3.9 ± 0.2				
M_oct_	4.8 ± 0.2			4.12^35^	
M_tet_	4.2 ± 0.3			4.20^35^	
M_total _(unit cell)	3.0 ± 0.7		3.1^36^	4.36^35^	3.3

Note: m_L_/m_S_ refers to the ratio of orbital and spin magnetic moment (m_L_ is the orbital magnetic moment and m_S_ is the spin magnetic moment). M_oct_ and M_tet_ are the total atomic magnetic moments (including the orbital and spin magnetic moment) of oct and tet Fe in the units of μ_B_ per atom. M_total_ is the total magnetic moments for Y_3_Fe_5_O_12_ in a unit cell.
