# Supplementary material for: A general way for quantitative magnetic measurement by transmitted electrons
Source: Sci Rep. 2016 Jan 4;6:18489. doi: 10.1038/srep18489 (PMC4698752; doi:10.1038/srep18489)
Supplement: Supplementary Information [file srep18489-s1.doc]

**Supplementary information**

**A general way for quantitative magnetic measurement by transmitted electrons**

Dongsheng Song^1^, Gen Li^1^,Jianwang Cai^2^ & Jing Zhu^1^,*

^1^ National Center for Electron Microscopy in Beijing, Key Laboratory of Advanced Materials (MOE) and The State Key Laboratory of New Ceramics and Fine Processing, School of Materials Science and Engineering, Tsinghua University, Beijing 100084, China.

^2^ Beijing National Laboratory for Condensed Matter Physics, Institute of Physics, Chinese Academy of Sciences, Beijing 100190, China

*Email: [jzhu@mail.tsinghua.edu.cn](mailto:jzhu@mail.tsinghua.edu.cn)

**Contact information for all authors**

Dongsheng Song: [todongsheng@126.com](mailto:todongsheng@126.com), 86-10-62787417

Gen Li: martinligen@163.com, 86-10-62787417

Jianwang Cai: [jwcai@aphy.iphy.ac.cn](mailto:jwcai@aphy.iphy.ac.cn), 86-10-82649094

Jing Zhu: [jzhu@mail.tsinghua.edu.cn](mailto:jzhu@mail.tsinghua.edu.cn), 86-10-62794026 **(corresponding author)**

1. **Analysis of octahedral enhancement**

The distribution of EMCD signals from octahedral and tetrahedral sites, and also their ratio are given separately as follows. It can be deduced from the figures that the area where the dynamical coefficients of octahedral site is larger than that of tetrahedral sites (ratio < 1), is corresponding to the enhancement of octahedral site. The result is consistent with the analysis in figure 2(d) that the area where the sign is inversed (around the white (black) areas in the upper (lower) half plane as shown in figure 2(d)) is almost corresponding to octahedral enhancement.


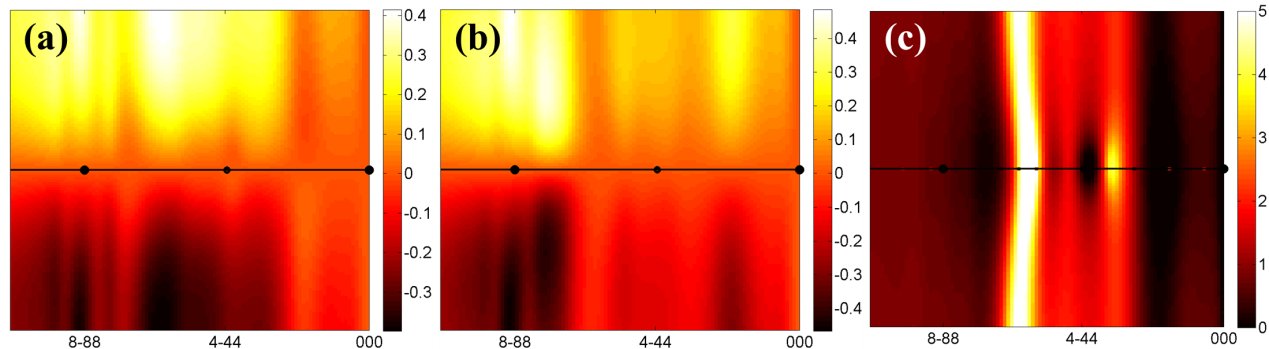


**Figure S1** Distribution of EMCD signals for tetrahedral sites (a), octahedral sites (b), and their ratio (c) in the diffraction plane with (8-88) planes strongly excited with the thickness of 45 nm and accelerating voltage of 300 kV.

**2. Simulation of EMCD signals with different planes excited**

The distribution of EMCD signals with the (004) and (2-20) planes strongly excited under the three-beam case are shown in figure S2. The intensity is about 5-10 times lower than that with (4-44) planes excited as shown in figure 2 in the manuscript.


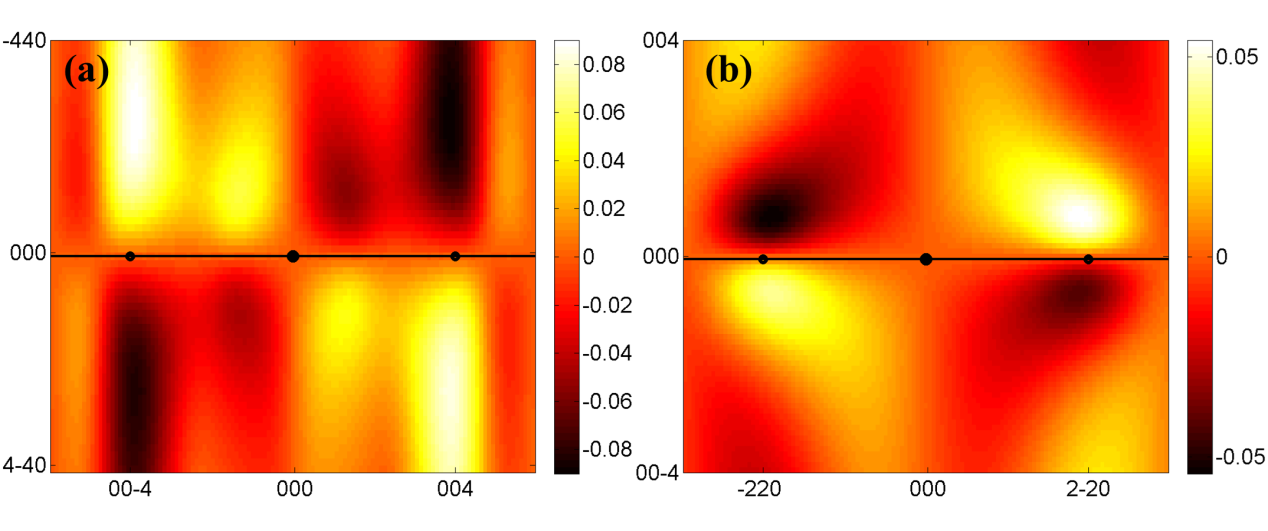


**Figure S2** Distribution of EMCD signals in the diffraction plane with (004) (a) and (2-20) (b) planes strongly excited under the three-beam case with the thickness of 45 nm and accelerating voltage of 300 kV.

The mapping of dynamical coefficients for octahedral and tetrahedral sites under two-beam, three-beam with (4-44) planes strongly excited and 2***θ_B_*** incident with (8-88) planes strongly excited are given as follows.


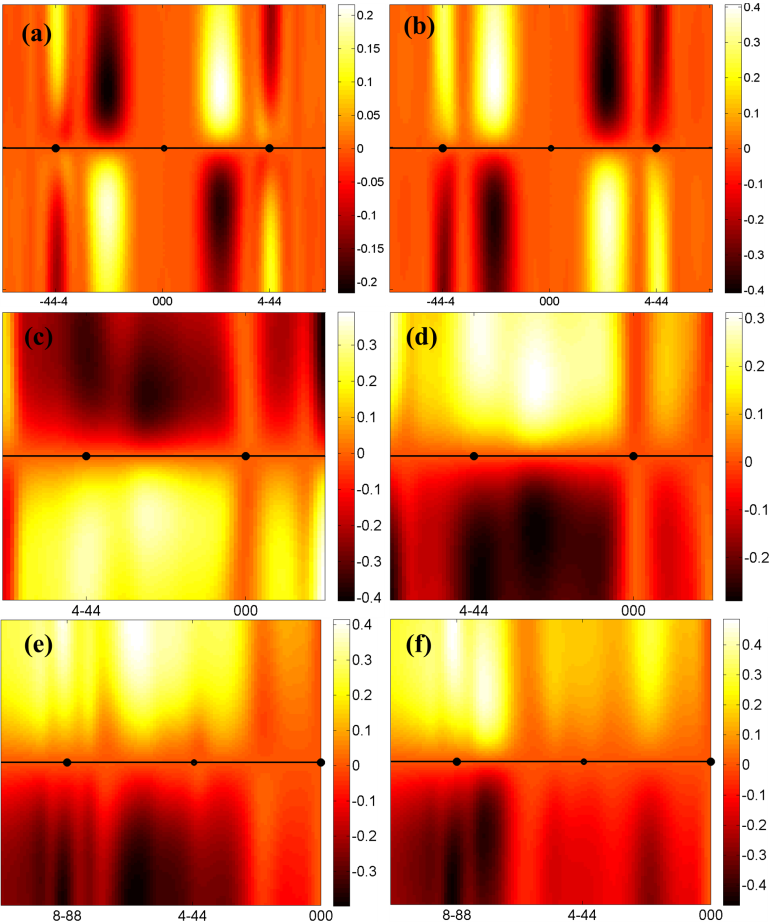


**Figure S3** Distribution of dynamical coefficients for tetrahedral sites (a,c,e), octahedral sites (b,d,f) in the diffraction plane under three-beam (a,b), two-beam (c,d), 2***θ_B_*** incident (e,f) case with the thickness of 45 nm and accelerating voltage of 300 kV.

**3. Asymmetry of EMCD diffraction geometry**

It has been ever emphasized that asymmetry caused by dynamical diffraction effects will lead to extrinsic non-zero nonmagnetic signals mixed with the magnetic signals, leading to artifacts in the EMCD signals. Therefore, much attention should be paid to the selection of detector position and area for spectra acquisition in the diffraction plane should not be only with high intensity of EMCD signals but also free from asymmetry^23,27^. The EELS spectra from the conjugate position can be expressed as equation (5) in the manuscript (Methods). To meet the requirement of symmetry, the dynamical coefficients for nonmagnetic signals should be equal for the “+” and “–” positons to make sure that the nonmagnetic signals are canceled out for pure EMCD signals. Then the magnitude of asymmetry is defined as the relative error of dynamical coefficients for nonmagnetic components,

$asymmetry=\frac{\alpha_{+}-\alpha_{-}}{\alpha_{+}+\alpha_{-}}$ (S1)

**α_+_** and **α-** are the dynamical coefficients as described in equation (6) and (7) for the plus and minus conjugate positions in the diffraction plane. Through the calculations of dynamical diffraction effects, the distribution of asymmetry for three kinds of diffraction geometry is shown in figure S4 ((a) for three-beam case, (b) for two-beam case, and (c) for 2θ_B_ incident). The areas with small values represent better cancelation of the nonmagnetic part and a purer EMCD signal. Similar to previous results^27^, the asymmetric part of nonmagnetic components is mainly localized around the Bragg spots, and the three-beam case with higher symmetry of crystallographic orientation has the smallest area being strongly affected. Nonetheless, there are still large areas with errors of negligible magnitude in the diffraction plane. In addition, these areas sometimes are corresponding to strong intensity of EMCD signals for the benefit of quantitative measurements. The detector positions with strong intensity and negligible asymmetry are selected in the experiments.


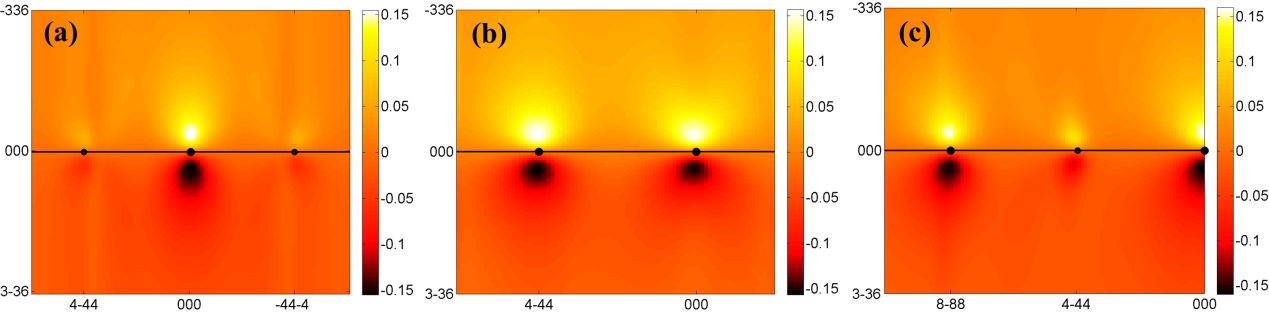


**Figure S4** Asymmetry part of nonmagnetic component in the diffraction plane. (a), (b), (c) are corresponding to the diffraction geometry of three-beam, two-beam, and 2θ_B_ incident, respectively.

**4. Errors analysis**

The estimation of the errors involves the noise in EMCD spectra, the measurement of sample thickness, and the calculations of theoretically obtained dynamical coefficients. The total error is obtained by adding them together.

For the noise in EMCD spectra, the standard errors for the magnetic parameters are calculated from a series of independent pairs of EMCD spectra as shown in table S1.The total errors of m_L_/m_S_ is almost totally contributed by this term, showing the great importance of SNR for quantitative EMCD technique. Besides, it also leads to a large error of magnetic moments of each atom. The measurement error of sample thickness is about ±1.7 nm determined by CBED and low-loss EELS. The dynamical coefficients corresponding to the thickness varied from 45.0 nm to 48.4 nm are calculated to give the error. However, its influence on magnetic parameters is negligible, for the change of dynamical coefficients in this range of thickness is very flat as shown in figure 2(c). The error of dynamical coefficients is difficult to be evaluated. The possible uncertainties in the imprecise knowledge of the experimental dynamical diffraction conditions will undoubtedly contribute to the error of magnetic parameters. These factors, such as orientation of sample and detector, convergence and collection angles, tilt angle from zone axis, partial coherence of the incident beam, the defects on the samples, et al will contribute to the uncertainty of dynamical coefficients, but only some of them currently can be determined. For example, the positons of detector and tilt angle. The center of detector is moved to right and left with 0.1G along the systematic row with respect to center of Thales circle with a diameter of 0.5G under the two-beam case during the calculations, which is close to position of collection aperture in the experiments. The change of dynamical diffraction coefficients is less than about 6.5%. For the tilt angle from [110] zone axis, we take the angle of 8.4°, 9.4°, 10.4° respectively to calculate the dynamical coefficients and the variation is less than 3%. As they all have an effect on the dynamical coefficients during the quantitative measurement, we use the 20% random error of dynamical coefficients to estimate their error (errors in table 1 take this case). Meanwhile, for its uncertainty, the random noise with maximum of ±10% and ±5% are also used to estimate the errors as shown in table S2. It is found that the errors for magnetic parameters are very sensitive to the value of random noise, especially the total magnetic moment. Therefore, the development of advanced mathematical method for signal extraction, which is free from calculation of dynamical parameters like multivariate curve resolution (MCR)^26,29,30,37^, is of great significance for further development of quantitative EMCD technique.

Table S1 Errors of different sources

| **Error sources** | ***m_L_/m_s_***  **(oct Fe)** | ***m_L_/m_s_***  **(tet Fe)** | ***M_oct,Fe_*** | ***M_tet,Fe_*** | ***M_tot_***  **(unit cell)** |
| --- | --- | --- | --- | --- | --- |
| **Noise** | 0.021 | 0.017 | 0.11 | 0.15 | 0.33 |
| **Thickness** | 0.000 | 0.000 | 0.01 | 0.01 | 0.04 |
| **Dynamical coefficients (20%)** | 0.003 | 0.004 | 0.15 | 0.18 | 0.65 |
| **Total error（20%）** | 0.021 | 0.018 | 0.19 | 0.24 | 0.73 |
| **Calculated value** | 0.06 | 0.08 | 4.8 | 4.2 | 3.0 |

Table S2 errors of dynamical coefficients with different random noise

| **Error sources** | ***m_L_/m_s_***  **(oct Fe)** | ***m_L_/m_s_***  **(tet Fe)** | ***M_oct,Fe_*** | ***M_tet,Fe_*** | ***M_tot_***  **(unit cell)** |
| --- | --- | --- | --- | --- | --- |
| **Dynamical coefficients (20%)** | 0.003 | 0.004 | 0.15 | 0.18 | 0.65 |
| **Dynamical coefficients (10%)** | 0.001 | 0.001 | 0.11 | 0.07 | 0.27 |
| **Dynamical coefficients (5%)** | 0.001 | 0.001 | 0.05 | 0.02 | 0.10 |
| **Total error（20%）** | 0.021 | 0.018 | 0.19 | 0.24 | 0.73 |
| **Total error（10%）** | 0.021 | 0.017 | 0.15 | 0.17 | 0.43 |
| **Total error（5%）** | 0.021 | 0.017 | 0.12 | 0.15 | 0.35 |
| **Calculated value** | 0.06 | 0.08 | 4.8 | 4.2 | 3.0 |

**5. Macroscopic magnetic measurements**

To estimate the magnetic moment of YIG, the superconducting quantum interference device (SQUID) was used to measure hysteresis [loop](javascript:void(0);) of YIG thin film (5mm*3mm*13.5μm) before prepared for TEM cross-sectional sample. The M-H loops of in plane and out of plane are shown in figure S5.


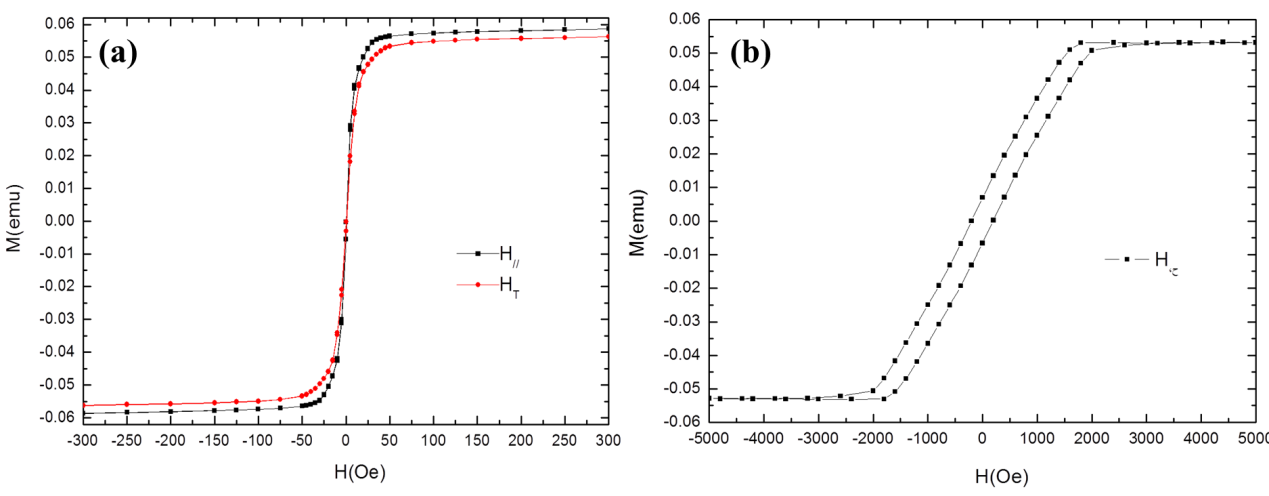


Figure S5 Magnetic hysteresis loops of YIG thin films measured by SQUID. (a) In plane M-H loops along longitudinal (perpendicular to large length) and transverse (parallel to large length) axis; (b) out of plane.

6. **Discussion about the convergence of dynamical calculation**

The convergence of dynamical coefficients is of necessity and it might have an effect on the results of quantitative magnetic measurements. Thus, we give a discussion about convergence of our calculations. For the calculated results in the manuscript, the calculation are based on the 5/5 beams for incoming/outgoing wave. To verify the convergence of the dynamical coefficients that are used in the quantitative analysis, new calculations with a large basis are performed. The calculations are only including the positions where the EMCD signals are acquired in the experiments, because these data are used during the quantitative magnetic measurements. Four different diffraction conditions are involved during the calculations: two-beam and three-beam case with (4-44) planes strongly excited, **2*θ*_B_** incident with (8-88) planes strongly excited for the octahedral and tetrahedral sites enhanced.

Two simple cases are preformed to verify the convergence. First, we take more beams along the systematical reflection axis. The 9/9 beams for incoming/outgoing wave along the reflection axis are considered to make a comparison with the original 5/5 beams during the calculation. The results are listed in table S3. It can be clearly seen that the difference is very small. Thus, we conclude that the 5/5 beams that we used in our original calculations are enough to guarantee the convergence of systematical reflection case. Secondly, we choose more beams to be included in the calculation to further verify the convergence. The 15/15 beams are automatically selected by the Bloch wave software by setting the maximum of ***w_g_***, which is not limited to the systematical reflection beams. Because our code is not simplified and efficient as MTAS in ref. 36, much time will be taken during the calculations. Similarly, the four different diffraction conditions are considered during the calculations as shown in table S3. Though more beams are considered during the calculations, the values of dynamical coefficient do not show obvious change. Besides, the new dynamical coefficients are also used to recalculate the magnetic parameters. The results almost do not change after we keep the significant figures.

These results show that the localized areas where the EMCD signals are acquired in our experiments are not significantly influenced by our new calculation results. But we believe the calculations with much larger basis will be of great significance when the EMCD signals in the diffraction plane are fully used to extraction the magnetic parameters, such as the energy filter mode.

Table S3 dynamical coefficient under different diffraction conditions

|  | **two-beam case** | | **three-beam case** | | **2*θ*_B_ case**  **(oct enhanced)** | | **2*θ*_B_ case**  **(tet enhanced)** | |
| --- | --- | --- | --- | --- | --- | --- | --- | --- |
|  | **tet** | **oct** | **tet** | **oct** | **tet** | **oct** | **tet** | **oct** |
| **5/5 beams** | 0.281 | -0.247 | 0.305 | -0.153 | -0.109 | -0.297 | -0.339 | -0.100 |
| **9/9 beams** | 0.281 | -0.248 | 0.299 | -0.155 | -0.107 | -0.295 | -0.341 | -0.097 |
| **15/15 beams** | 0.280 | -0.250 | 0.306 | -0.151 | -0.103 | -0.300 | -0.337 | -0.099 |

Note: **tet** and **oct** represent the dynamical coefficients for tetrahedral and octahedral sites, respectively. The dynamical coefficients are calculated by averaging the values at the positons where the EMCD signals are acquired in the experiments. The diffraction conditions include the two-beam case, three-beam case, **2*θ*_B_** incident case with the octahedral and tetrahedral sites enhanced, respectively.

**Reference**

37. Tatsumi, K. & Muto, S. Local electronic structure analysis by site-selective ELNES using electron channeling and first-principles calculations. J. Phys.: Condens. Matter 21, 104213 (2009).
